# Abiraterone and MDV3100 inhibits the proliferation and promotes the apoptosis of prostate cancer cells through mitophagy

**DOI:** 10.1186/s12935-019-1021-9

**Published:** 2019-12-10

**Authors:** Jingli Han, Junhua Zhang, Wei Zhang, Dalei Zhang, Ying Li, Jinsong Zhang, Yaqun Zhang, Tongxiang Diao, Luwei Cui, Wenqing Li, Fei Xiao, Ming Liu, Lihui Zou

**Affiliations:** 1 The MOH Key Laboratory of Geriatrics, Beijing Hospital, National Center of Gerontology, Beijing, 100730 People’s Republic of China; 2 Department of Pathology, Beijing Hospital, National Center of Gerontology, Beijing, 100730 People’s Republic of China; 3 Department of Urology, Beijing Hospital, National Center of Gerontology, Beijing, 100730 People’s Republic of China; 4 Clinical Biobank, Beijing Hospital, National Center of Gerontology, Beijing, 100730 People’s Republic of China

**Keywords:** Prostate cancer, Mitophagy, Abiraterone, MDV3100

## Abstract

**Background:**

Abiraterone and MDV3100 are two effective anticancer agents for prostate cancer, however, the mechanism of their downstream action remains undefined.

**Methods:**

A dual fluorescent biosensor plasmid was transfected in LNCaP cells to measure mitophagy. The DNA of LNCaP cells was extracted and performed with quantitative real-time PCR to detect mitochondrial DNA copy number. JC-1 staining was utilized to detect the mitochondrial membrane potential and electron microscope was performed to analyze mitochondrial morphology. Moreover, the protein levels of mitochondrial markers and apoptotic markers were detected by western blot. At last, the proliferation and apoptosis of LNCaP cells were analyzed with CCK-8 assay and flow cytometry after abiraterone or MDV3100 treatment.

**Results:**

Mitophagy was induced by abiraterone and MDV3100 in LNCaP cells. The low expression level of mitochondrial DNA copy number and mitochondrial depolarization were further identified in the abiraterone or MDV3100 treatment groups compared with the control group. Besides, severe mitochondria swelling and substantial autophagy-lysosomes were observed in abiraterone- and MDV3100-treated LNCaP cells. The expression of mitochondria-related proteins, frataxin, ACO2 and Tom20 were significantly downregulated in abiraterone and MDV3100 treated LNCaP cells, whereas the expression level of inner membrane protein of mitochondria (Tim23) was significantly upregulated in the same condition. Moreover, the proliferation of LNCaP cells were drastically inhibited, and the apoptosis of LNCaP cells was increased in abiraterone or MDV3100 treatment groups. Meanwhile, the addition of mitophagy inhibitor Mdivi-1 (mitochondrial division inhibitor 1) could conversely elevate proliferation and constrain apoptosis of LNCaP cells.

**Conclusions:**

Our results prove that both abiraterone and MDV3100 inhibit the proliferation, promote the apoptosis of prostate cancer cells through regulating mitophagy. The promotion of mitophagy might enhance the efficacy of abiraterone and MDV3100, which could be a potential strategy to improve chemotherapy with these two reagents.

## Background

Prostate cancer (PCa) is one of the most common carcinomas in men with promptly increasing incidence rate and high mortality in the past decade [1]. Abiraterone acetate, the precursor of abiraterone, is an androgen biosynthesis inhibitor which is used in the treatment of prostate cancer, specifically in combination with castration and prednisone for the treatment of metastatic castration-resistant prostate cancer (mCRPC) and metastatic high-risk castration-sensitive prostate cancer (mCSPC) [2, 3]. Abiraterone also antagonizes androgen receptor (AR), which plays a crucial role in the progression of prostate cancer [4, 5]. MDV3100, the second-generation antiandrogen enzalutamide, is another effective drug for treatment of prostate cancer. MDV3100 binds directly to AR and competitively inhibits androgen binding, thus hampers AR nuclear translocation and AR-mediated DNA binding [4, 6]. MDV3100 also has a therapeutic effect on CRPC with elevated AR expression and nonmetastatic CRPC with increased prostate-specific antigen (PSA) level [7, 8].

Over the past decade, many studies have demonstrated that autophagy and mitophagy were frequently induced by anticarcinogens under the stimulation of multiple forms of cellular stresses [9, 10]. Autophagy is a conserved homeostatic pathway that degrades damaged proteins or organelles through the formation of autophagic lysosomes with a two-membrane compartment [11]. Mitophagy is a type of autophagy, which maintains the quality of mitochondria and maintains the health of cells by scavenging aged and damaged mitochondria [12, 13]. Moreover, mitophagy would further activate autophagy which is important for the regulation of cancer progression and helps to determine how cancer cells respond to anticancer therapies [9, 14]. Therefore, the promotion or suppression of mitophagy might be a potential strategy for clinical therapy. Although the roles of abiraterone and MDV3100 in anti-tumor activity have been reported [2, 14], the relationship between them and mitochondrial functions, especially mitophagy, in prostate cancer cells is still unclear.

In this paper, we used a plasmid that can co-express mitochondrial associated protein subunit VIII of cytochrome c oxidase (COX8) [15], non-pH-sensitive discosoma red fluorescent protein (DsRed), and pH-sensitive green fluorescent protein (pHluorin), which can be used to detect mitophagy in cells because the intracellular pH was reduced when mitophagy occurs [16]. The combination of DsRed and pHluorin was called Rosella, while COX8 is one of the mitochondria-specific proteins. The intensity of pHluorin changed due to intracellular pH in eukaryotic cells, while the intensity of DsRed did not. The constructed plasmid used to measure mitophagy was named as pDsRed-Mito-Rosella, which determines mitophagy with reduced green to red fluorescent ratio. Human prostate adenocarcinoma cells lymph node carcinoma of the prostate (LNCaP) with high expression of AR has long been considered as the closest model for prostate cancer study, and its proliferation depends on the activation of androgen receptor signaling pathway. It can be used to investigate the downstream mechanism of abiraterone and MDV3100 in the treatment of prostate cancer [17]. Here, we found that abiraterone and MDV3100 induced mitophagy measured via the plasmid pDsRed-Mito-Rosella biosensor, which was consistent with further identification of canonical mitophagy, mitochondrial membrane depolarization, mitochondria swelling, and decreased expression of crucial mitochondrial proteins such as ACO2, frataxin and ATP synthetase. Moreover, the proliferation of prostate cancer cells was inhibited and the apoptosis of prostate cancer cells was promoted, both of which were converted by mitophagy inhibitor Mdivi-1.

## Materials and methods

### Cell culture and transfection

LNCaP cells purchased from American Type Culture Collection (ATCC) were cultured in RPMI-1640 medium (Gbico, Thermo Fisher Scientific, USA) supplemented with 10% fetal bovine serum (HyClone Inc., Utah, USA) and incubated at 37 °C with 5% CO_2_. The exponentially growing LNCaP cells were treated with 20 nmol/L abiraterone (Aladdin Industral Corp., Shanghai, China) for 24 h or 10 nmol/L MDV3100 (MedChem Express, Shanghai, China) for 48 h or 20 μmol/L mdivi-1(MedChem Express, Shanghai, China). The plasmids, pDsRed-Rosella-LC3, pDsRed-Mito-Rosella and pDsRed-NC were constructed in our laboratory referred the study of Sargsyan et al. [16] and some alterations were made. Plasmid Rosella-LC3 consists of the complete open reading frame (ORF) of human LC3B (Map1lc3b) joined in frame with the 3′ end of the Rosella ORF (coding the C-terminus) without a stop codon. Plasmid Mito-Rosella consists of the subunit VIII of cytochrome c oxidase (COX8) ORF without a stop codon fused in frame with the 5′ end of the Rosella ORF (coding the N-terminus). LNCaP cells were seeded in 6-well dish and transfected with plasmid (2.5 μg/well) using lipofectamine 3000 reagent (Invitrogen, Thermo Fisher Scientific, USA) in accordance with standard experimental procedure.

### Confocal microscopy assay

To determine mitophagy or autophagy in LNCaP cells, they were exposed to abiraterone or MDV3100 after 48 h transfection, then the nuclei were stained with 1 μg/mL DAPI (Thermo Fisher Scientific, USA) for 30 min. The fluorescence changes in response to drugs or vehicle treatment were observed and measured in more than 25 fields with a confocal fluorescence microscope (Nikon, Japan) at 10× 40 magnification. Vehicle-treated cells served as a control.

### Quantitative real-time PCR (qPCR)

Total DNA was isolated from LNCaP cells using TIANcombi DNA Lyse&Det PCR Kit (TIANGEN Biotech Corp., Beijing, China). Quantitative real-time PCR was performed to quantify mitochondrial DNA (mtDNA) copy number and the internal control gene β-globin using SYBR^®^ Premix Ex TaqTM II (Takara Biotech Co., Dalian, China) and the specific primers: mtDNA 172- 5′-AGGACAAGAGAAATAAGGCC-3′ (forward), 5′-TAAGAAGAGGAATTGAACCTCTGACTGTAA-3′ (reverse) and β-globin 5′-GTGCACCTGACTCCTGAGGAGA-3′ (forward), 5′-CCTTGATACCAACCTGCCCAG-3′ (reverse). The conditions of PCR reaction were as follows: one cycle of 95 °C for 30 s, 40 cycles of 95 °C for 5 s, and 60 °C for 30 s. Each reaction was repeated three times. The expression level of mtDNA was determined by relative quantitative CT value of gene expression with 2^−∆∆CT^ method.

### Mitochondrial membrane potential (mΔψ) detection

Mitochondrial membrane potential was measured using the cationic dye, JC-1 (5′,6,6′-tetrachloro-1,1′,3,3′-tetraethylbenzimidazolylcarbocyanine iodide), and the mitochondrial membrane potential disrupter, CCCP (carbonyl cyanide 3-chlorophenylhydrazone) as a positive treatment. JC-1 can potential-dependently accumulate in mitochondria indicated by a fluorescence change from green to red. Therefore, the decline in the red/green fluorescence intensity ratio can indicate mitochondrial depolarization. After LNCaP cells were treated with abiraterone or MDV3100 as described above, they were collected and incubated with 1 μmol/L JC-1 (MitoProbe™ JC-1 Assay Kit, Thermo Fisher Scientific, USA) and exposed to darkness at 37 °C for 15 min. MΔψ was detected by flow cytometry with Accuri C6 PLUS flow cytometer (BD Biosciences, San Jose, CA, USA).

### Protein isolation and western blot

The total proteins were extracted from the treated cells with RIPA buffer (Beyotime Biotech, Haimen, China) containing protease inhibitor cocktail (Thermo Fisher Scientific, USA). The concentrations of total protein lysates were determined by the enhanced bicinchoninic acid (BCA) protein quantification assay kit (Zomanbio, Beijing, China). Next, proteins (40 μg per well) were used for 12% sodium dodecyl sulphate–polyacrylamide gel electrophoresis. Then, the proteins were transferred to polyvinylidene fluoride (PVDF) membranes which then were blocked with 5% skimmed milk. All kinds of antibodies were diluted by antibody dilution solution (Beyotime Biotech, Haimen, China) and were incubated under the manual instruction.

### Transmission electron microscopy (TEM)

The treated cells were prepared with cold phosphate buffer saline (PBS), trypsinized and centrifuged to accumulate the cells into a mass. The cell clusters were fixed with fresh 2.5% glutaraldehyde in 0.1 mol/L phosphate buffer (pH 7.4) at 4 °C overnight. LNCaP cells were washed with cold PBS and incubated in 1% osmium tetroxide for 1 h at 4 °C. The cell clusters then underwent a series of dehydration and embedded in epon resin. The samples were cut into ultrathin sections and stained with 2% uranyl acetate and photographed with a JEM-1400 plus transmission electron microscope (JEOL Ltd., Tokyo, Japan) to analyze the cellular ultrastructure.

### Cell proliferation analysis

LNCaP cells (5000 per well) were seeded in 96-well plates one day before treatment with abiraterone and MDV3100. After drug treatments, CCK-8 (Cell Counting Kit-8, Dojindo Molecular Technologies, Inc., Rockville, USA) was added to each well, and incubated in darkness at 37 °C for 2 h. The absorbance of 96-well plate was measured at room temperature by microporous plate reader at 450 nm. Then the cell proliferation ability was measured every 24 h, and the absorbance was normalized to 0 h.

### Flow cytometric (FCM) analysis of cell apoptosis

The treated LNCaP cells was explored with Annexin V-FITC/PI (Zoman Biotech. Beijing, China) according to the manufacturer’s protocol. 1 × 10^5^ cells were suspended in 500 μL Annexin V-FITC binding buffer containing 5 μL Annexin V-FITC and 10 μL propidium iodide. After incubated in darkness for 30 min, the cells were subjected to a FCM assay performed with Accuri C6 PLUS flow cytometer (BD Biosciences, San Jose, CA, USA).

### Statistical analysis

Data were analyzed using the SPSS 20.0 statistical software package. The data were presented as the mean × standard deviation (S.D.) of at least three independent experiments. Unpaired *t* test was used to determine significant differences between the treated and control groups, and a *p* < 0.05 was considered statistically significant.

## Results

### Abiraterone and MDV3100 both activate mitophagy in LNCaP cell

**Fig. 1:**
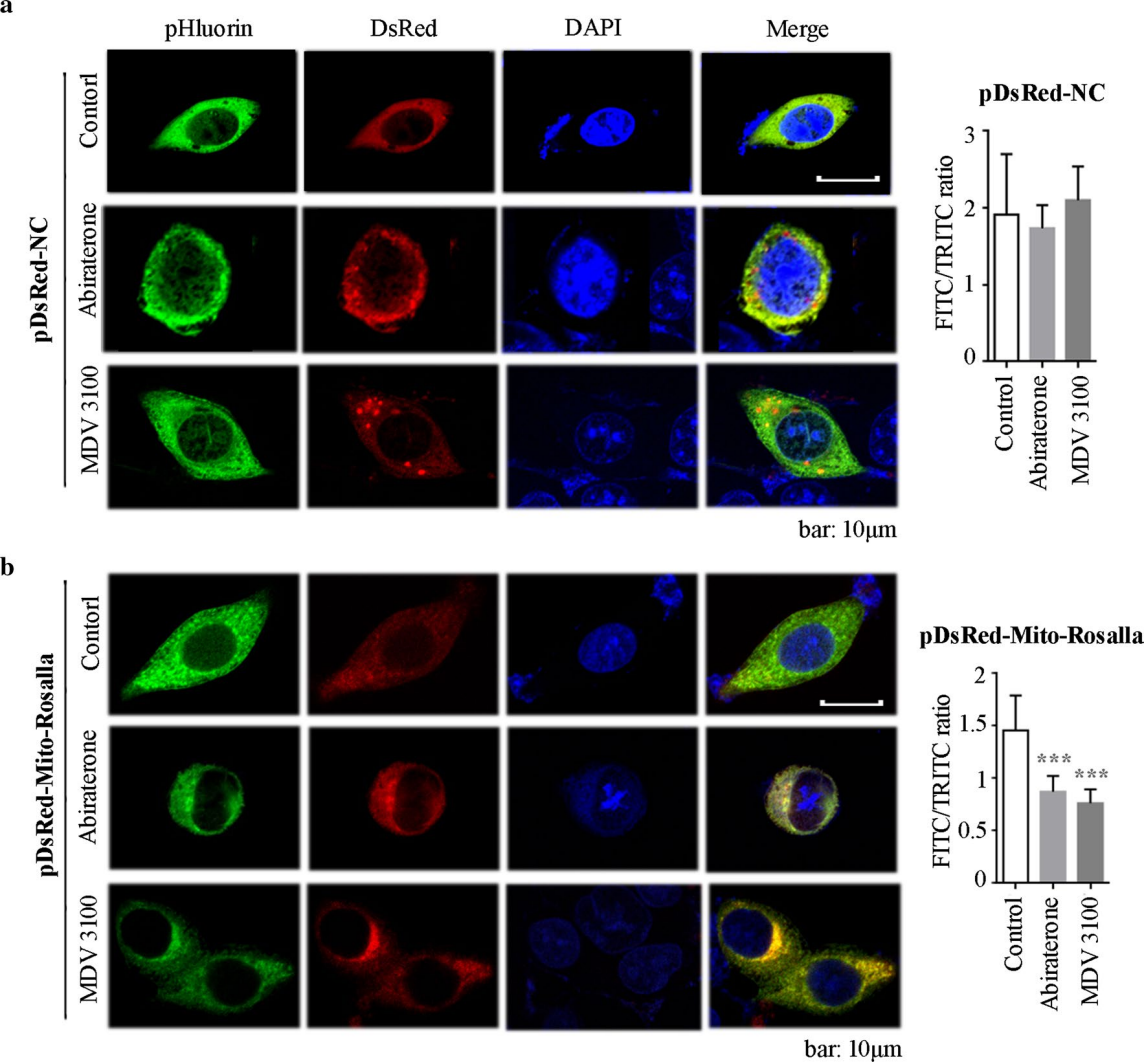
Abiraterone and MDV3100 induced mitophagy in LNCaP cells. **a** Representative micrographs of LNCaP cells transiently transfected with pDsRed-NC expression plasmids. The cells were treated with vehicle alone (control), abiraterone, or MDV3100. **b** Representative micrographs of green and red channel fluorescence of Mito-Rosella transiently transfected cells following treatments described above for **a**. The merged panel shows overlap of fluorescence from the pHluorin, DsRed and DAPI. The green/red fluorescence ratio of single cell under the above conditions was quantitatively measured. Error bars represent mean ± S.D. of ratios for n = 25 cells per condition. The experiments were performed three times, and a representative result is shown above; ***p < 0.001 versus control, based on unpaired *t*-test. NC: normal control; DAPI: 4′,6-diamidino-2-phenylindole

In the DsRed and pHluorin combination dual fluorescent biosensors, COX8 can specifically label mitochondria in LNCaP cell. In pDsRed-NC transfection groups, the intensity of red and green fluorescent protein did not change after abiraterone and MDV3100 treatment, while in pDsRed-Mtio-Rosella transfection groups, abiraterone and MDV3100 treatment remarkably decreased the green fluorescence intensity with a significant drop of green to red fluorescent ratio (Fig. 1).

Besides, abiraterone and MDV3100 treatment groups displayed accumulation of fluorescence cellular location, while the fluorescence in control group has a diffuse localization. Moreover, drug-treated groups had undergone different levels of nuclear fragmentation and nuclear shrinkage, providing evidence for abiraterone- and MDV3100-induced apoptosis in LNCaP cells.

### Mitochondrial DNA copy number, mitochondrial membrane potential (mΔψ) and morphology detection in abiraterone- and MDV3100-treated LNCaP cells

**Fig. 2:**
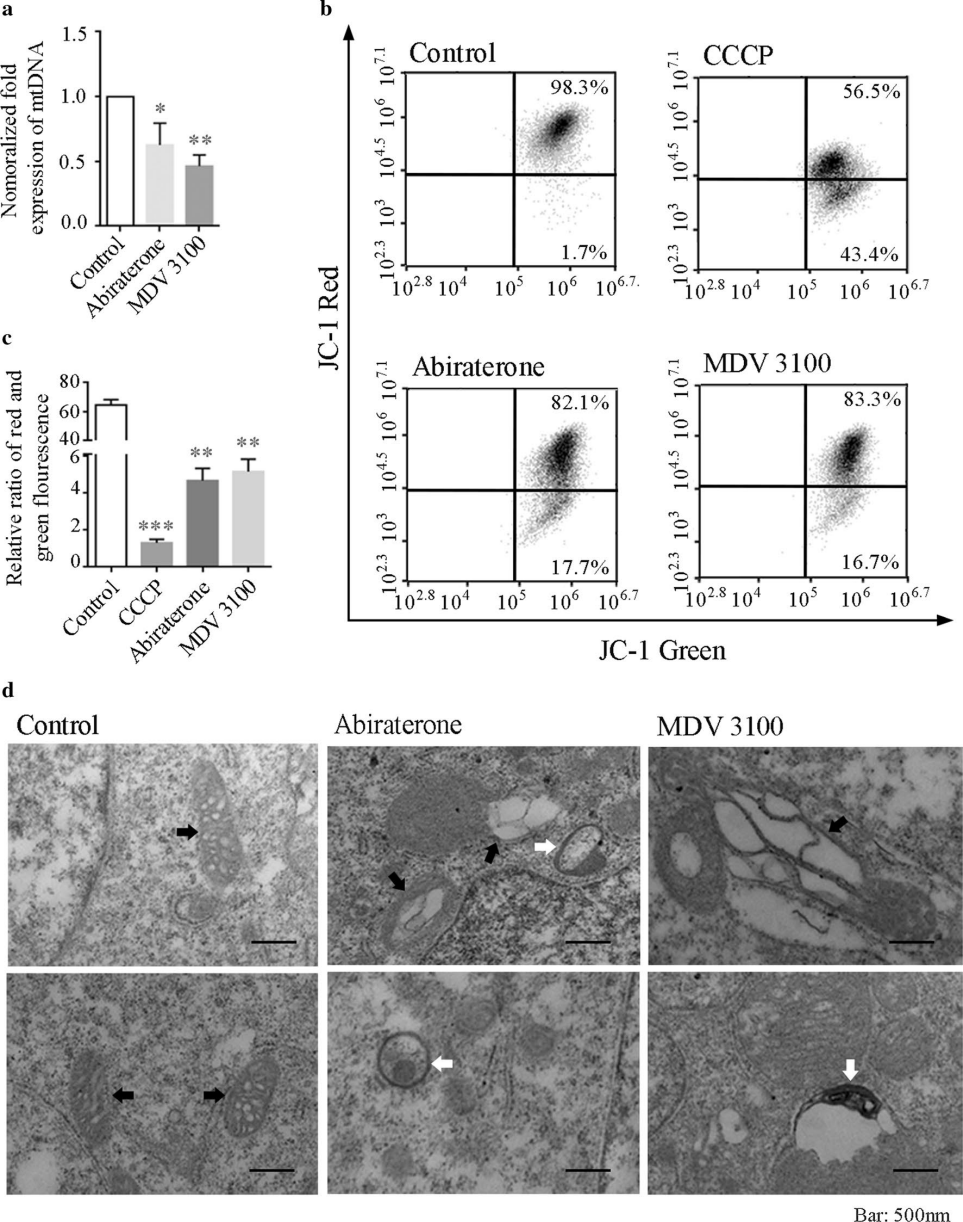
Effects of abiraterone and MDV3100 on mtDNA, mΔψ and morphology in LNCaP cell. **a** Detection of mitochondrial DNA copy number. Error bars represent mean ± S.D. of three independent experiments; *p < 0.05, **p < 0.01 versus control, based on unpaired *t*-test. **b**, **c** Mitochondrial membrane potential (mΔψ) detection. The representative dot plots of JC-1 fluorescence in the LNCaP cells treated with 20 nmol/L abiraterone for 24 h or 10 nmol/L MDV3100 for 48 h. 50 M/L CCCP for 5 min working as positive control of the assay. Error bars represent mean ± S.D. of three independent experiments; n ≥ 10,000 cells/experiment. *p < 0.05, **p < 0.01, ***p < 0.001versus control, based on unpaired *t*-test. **d** Mitochondrial morphology analysis. Electron micrographs show swelling mitochondria and autophagosomes induced by abiraterone and MDV3100. The white arrows in the electron micrographs represent autophagosomes, the black ones represent swelling mitochondria in abiraterone and MDV3100 treatment groups and represent healthy mitochondria in control group. Scale bar, 500 nm. The experiments were performed three times, and a representative result is shown above

Further, we need to confirm whether abiraterone and/or MDV3100 were involved in mitochondrial damage. Mitochondrial DNA is quite unstable and fragile without protection like nuclear membrane, and to some extent reflects the state of the mitochondria [18, 19]. In the current study, the copy number of mtDNA decreased significantly in abiraterone- and MDV3100-treat LNCaP cells compared to vehicle indicating mtDNA damage was caused by both of them (Fig. 2a).

The state and function of mitochondria can be evaluated by the permeability of the mitochondrial membrane, the capacity of the mitochondrial proton pump, and the activity of the electron transport system, such as mΔψ [20–23]. In order to evaluate the effects of these two drugs on mitochondria, we used the specific mitochondrial probe JC-1 to detect the effects of these two drugs on mΔψ of LNCaP cells. At the same time, we used CCCP as a positive control to induce the decrease of mitochondrial membrane potential. Consequently, mitochondrial depolarization was indicated by a decrease in the red/green fluorescence intensity ratio in drug-treated LNCaP cells, which is dependent on the membrane potential (Fig. 2b, c). Moreover, severe mitochondria swelling and the amount of autophagy-lysosomes were observed in abiraterone- and MDV3100-treated LNCaP cells through electron micrographs (Fig. 2d).

### Western blot analysis of mitochondrial proteins and mitophagy markers

**Fig. 3:**
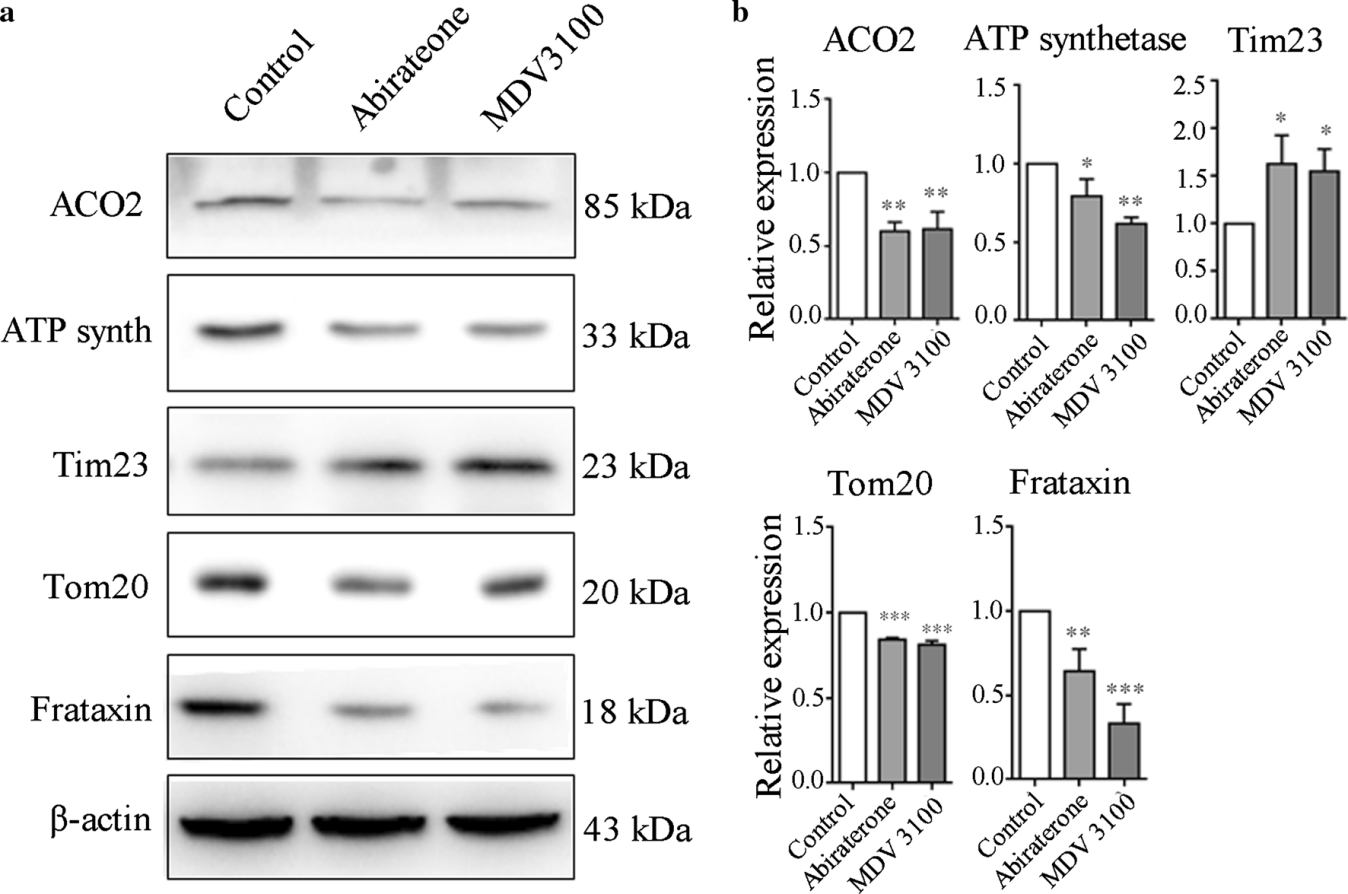
Effects of abiraterone and MDV3100 on expression of mitochondrial marker proteins. **a** Western blotting analysis for expression of the ACO2, ATP synthetase, Tim 23, Tom 20 and frataxin. LNCaP cells were treated with MDV3100 or abiraterone as designed. β-actin served as a loading control. **b** The grouping column diagram present the relative expression of mitochondrial-related proteins normalized to β-actin in LNCaP cells after treatment with abiraterone or MDV3100. Error bars represent mean ± S.D. of three independent experiments and a representative result is shown above, *p < 0.05, **p < 0.01; ***p < 0.001 versus control, based on unpaired *t*-test. ACO2: aconitase2; ATP synth: ATP synthetase; Tim23: the intimal translocation enzyme of mitochondria; Tom20: the adventitia translocation enzyme of mitochondria

Next, western blot analysis of mitochondrial marker proteins will determine the extent of mitophagy. It is well known that the decrease of mΔψ is accompanied by the free passage of small molecules through the mitochondrial membrane, thus depriving the ATP synthase of its ability to provide sufficient energy, finally leading to cell death [23]. Consistent with this, our data showed the expression of ATP synthase was significantly downregulated in abiraterone and MDV3100 treatment LNCaP cells. In addition, the expression of crucial mitochondria-related proteins, frataxin, aconitase 2 (ACO2) and the translocase of the outer membrane (Tom20) were apparently downregulated in LNCaP cells following treatment of abiraterone and MDV3100. Whereas the expression level of the translocase of the inner membrane (Tim23) was significantly upregulated in the same condition (Fig. 3a, b).

### Abiraterone and MDV3100 also induced autophagy in LNCaP cells

**Fig. 4:**
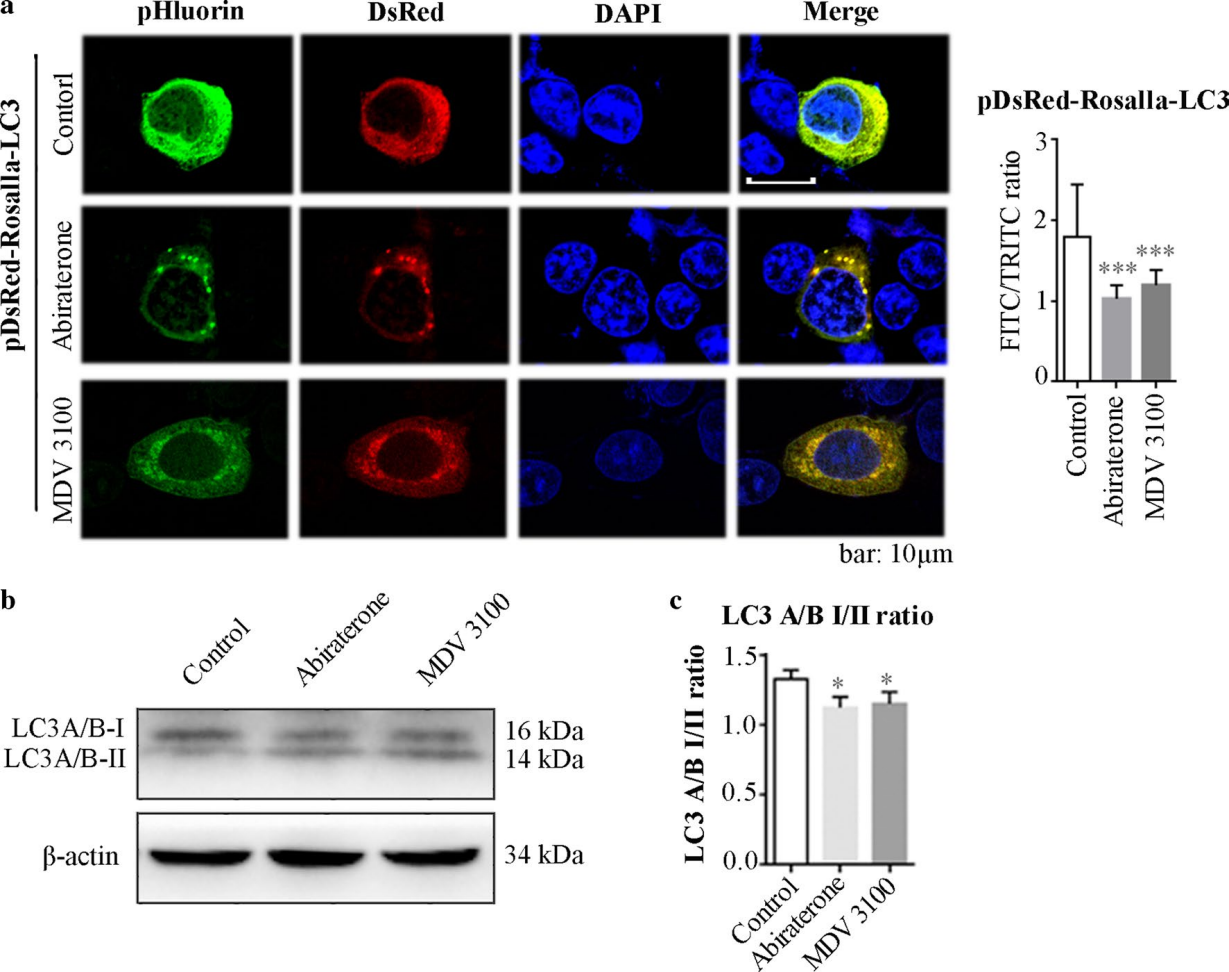
Abiraterone and MDV3100 induced autophagy in LNCaP cells. **a** Representative micrographs of LNCaP cells transiently transfected with expression plasmids for Rosella-LC3 and treated with abiraterone and MDV3100. The green to red fluorescence ratio was measured in single cell under exposure described above. Error bars represent mean ± S.D. of ratios for n = 25 cells per condition. ***p < 0.001 versus control, based on unpaired *t*-test. **b** Western blotting show effects of abiraterone and MDV3100 on expression of endogenous LC3 lipidation. β-actin was served as a loading control. **c** Grouped column diagram present the ratio of LC3A/B-I to LC3A/B-II quantified after normalization with β-actin in LNCaP cells with abiraterone or MDV3100 treatment. Error bars represent mean ± S.D. of three independent experiments and a representative result is shown above, *p < 0.05 versus control, based on unpaired *t*-test. DAPI: 4′,6-diamidino-2-phenylindole; LC3: light chain 3

Since mitophagy requires autophagy, we have also analyzed autophagy (i.e., Rosella-LC3) after abiraterone and MDV3100 treatment as well as mitophagy. In vehicle-treated cells, the green/red double labeled Rosella-LC3 showed a punctate diffuse localization throughout the cytoplasm. While abiraterone and MDV3100 treatment resulted in the aggregation of Rosella-LC3 with significantly reduced green fluorescence around the nucleus, indicating the redistribution of autophagosomes in low pH environment (Fig. 4a). Moreover, the relative expression level of autophagic associated LC3A/B-I was decreased, the expression of LC3A/B-II was increased, and the ratio of LC3A/B-I to LC3A/B-II was significantly decreased after abiraterone and MDV3100 treatment in LNCaP cells (Fig.4b).

### Abiraterone and MDV3100 significantly inhibit proliferation, elevated apoptosis via the regulation of mitophagy in LNCaP cells

**Fig. 5:**
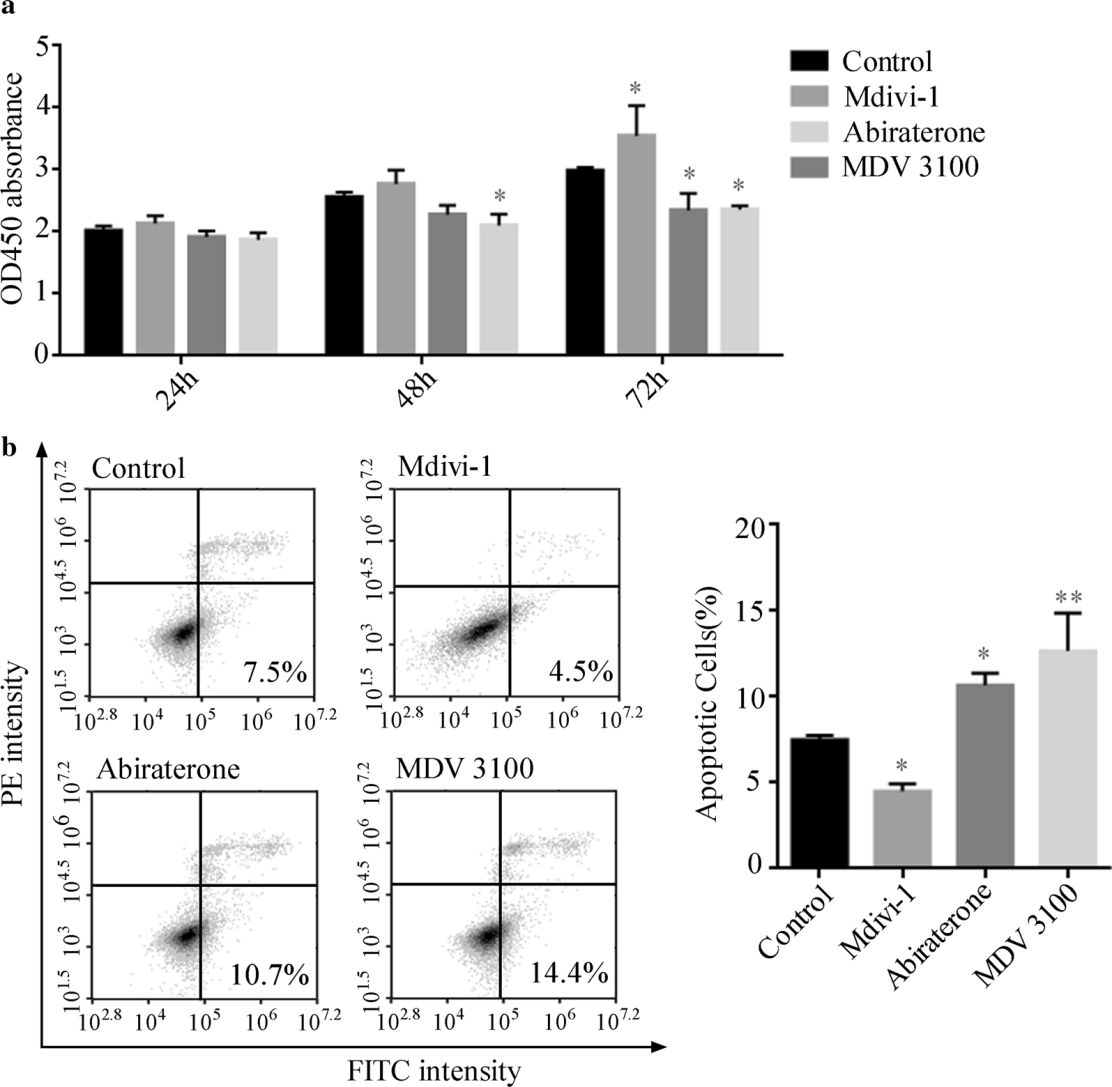
Abiraterone and MDV 3100 mediated cell proliferation and apoptosis in LNCaP cells through regulating mitophagy. **a** The blockade of mitophagy and abiraterone- and MDV3100-mediated inhibition of proliferation in LNCaP cells. LNCaP cells were treated with mdivi-1 or abiraterone or MDV3100 for 24 h, 48 h and 72 h, respectively. The absorbance values with CCK-8 were detected at 450 nm. Error bars represent mean ± S.D. of three independent experiments, *p < 0.05, **p < 0.01, ***p < 0.001 versus control, based on *t*-test. **b** Representative dot plots of Annexin-V-FITC and PE stained by propidium iodide (PI) in the LNCaP cells following treatments described above for A.MDV3100 Error bars represent mean ± S.D. of three independent experiments, *p < 0.05, **p < 0.01 versus control, based on *t*-test. Mdivi-1: Mitochondrial division inhibitor 1

In addition, effects on tumor cell proliferation, apoptosis and migration in LNCaP cells with abiraterone or MDV3100 exposure were observed. Our study showed the proliferation of LNCaP cells was inhibited significantly at the 72 h treatment time points in abiraterone or MDV3100 treated groups. However, the proliferation of LNCaP cells conversely increased at 72 h in Mdivi-1 treated group as showed in Fig. 5a.

A large number of studies have shown that mitophagy are closely related to apoptosis [24–26]. Consistent with nuclear fragmentation observed in Fig. 1, we observed that the apoptotic LNCaP cells significantly increased following both abiraterone (10.5%) and MDV3100 (14.4%) treatment, while decreased with Mdivi-1 exposure, compared to the vehicle (Fig. 5b).

## Discussion

In this study, we identified the potential mechanisms involved in the induction of mitophagy by abiraterone and MDV3100 treatments and confirmed the regulatory function of mitophagy on the anticancer effects of both drugs in LNCaP cell.

Recently, accumulating studies have revealed that reduced mitochondrial membrane potential, increased mitochondrial fragmentation, abnormal mitochondrial swelling and upregulated autophagic proteins, together result in mitophagy [20, 24, 26], which is important in various types of cancer. For instance, the proliferation of colon cancer cells was remarkably inhibited through mitophagy induced by mitochondrial-target anticancer agent [20], and the cellular apoptosis of colorectal cancer was enhanced by inhibition of mitophagy activity [20, 27]. Among these findings, mitophagy seems to play two opposite roles in cancer, the promotion of cancer progression and the inhibition of cancer development. Our study demonstrated the positive regulation of mitophagy on the antitumor activity of abiraterone and MDV3100 in LNCaP cells.

Our study suggested that abiraterone and MDV3100 induced mitophagy in LNCaP cell powerfully supported by apparent changes of mitochondrial morphology and decreased mitochondrial membrane potential. Therefore, the oxidized and damaged proteins are phagocytized by mitochondria with lower membrane potential, promoting the production of autophagosomes, which are then eliminated by mitophagy. The activation of mitochondrial permeability transition (MPT) enables small molecular weight proteins to pass freely through the mitochondrial membrane, depriving ATP synthetase of its ability to provide sufficient energy.

On the other hand, abiraterone and MDV3100 can also induce disordered expression of crucial mitochondrial proteins in LNCaP cells. Mitochondria must import all the proteins needed for their function through the adventitia translocation enzyme (TOM) and the intimal translocation enzyme (TIM). The decrease of TOM and the increase of TIM we detected may affect the material transport of mitochondria and lead to mitochondrial dysfunction. In addition, the decreased expression of ATP synthase, frataxin and ACO2, all of which were closely related to energy production, were detected after the induction of abiraterone and MDV3100. Mitochondrial ATP synthase located in mitochondrial inner membrane catalyzes ATP synthesis, utilizing an electrochemical gradient of protons across the inner membrane during oxidative phosphorylation. Frataxin appears to function in some capacity for iron-storage for the mitochondria, also function as an activator of oxidative phosphorylation to increase mitochondrial membrane potential and elevate cellular ATP [28]. Therefore, alterations of the mitochondrial membrane potential that stimulated by abiraterone and MDV3100 treatment play an essential role in the regulation of mitophagy. Aconitase 2 is encoded in the nucleus while functions in the mitochondrion and catalyzes the interconversion of citrate to isocitrate in the second step of the TCA cycle [29]. Thus the dysfunction of these mitochondrial function related proteins aggravates the energy deficiency and eventually led to cell death.

Further, in order to clarify the function of abiraterone and MDV3100 on LNCaP cells through mitophagy, we used mitophagy inhibitor Mdivi-1 in LNCaP cells. The results showed that proliferation of LNCaP cells were enhanced and the apoptosis was inhibited after blocking mitophagy. Therefore, abiraterone and MDV3100 can affect the proliferation and apoptosis of LNCaP cells through mitophagy.

## Conclusions

Taken together, mitophagy may not be the direct cause of cell death, but it does represent an adaptive response to chemotherapeutic stress that could restricts cell function by promoting apoptosis, inhibiting cellular proliferation. Therefore, the promotion of mitophagy might enhance the efficacy of abiraterone and MDV3100, which could be a potential strategy to improve the efficacy of chemotherapy with these two reagents.

## Data Availability

The datasets used and analyzed during the current study are available from the corresponding author on reasonable request.

## Abbreviations

PCa: prostate cancer
mCRPC: metastatic castration-resistant prostate cancer
mCSPC: castration-sensitive prostate cancer
LNCaP: lymph node carcinoma of the prostate
ACO2: aconitase 2
Tom20: the translocase of the outer membrane
Tim23: inner membrane protein of mitochondria
CCCP: carbonyl cyanide 3-chlorophenylhydrazone
Mdivi-1: mitochondrial division inhibitor 1
ATCC: American Type Culture Collection
ORF: open reading frame
COX8: the subunit VIII of cytochrome c oxidase
LC3: light chain 3
DsRed: discosoma red fluorescent protein
pHluorin: pH-sensitive green fluorescent protein
MPT: mitochondrial permeability transition
